# Comparison of the Simplexa GBS Direct and ARIES GBS assays for the detection of *S. agalactiae* in broth-enriched swab specimens

**DOI:** 10.1128/spectrum.04164-23

**Published:** 2024-03-05

**Authors:** Lavannya Sabharwal, Matthew L. Faron, Blake W. Buchan

**Affiliations:** 1 Department of Pathology, The Medical College of Wisconsin, Milwaukee, Wisconsin, USA; 2 Children’s Wisconsin, Milwaukee, Wisconsin, USA; University of Maryland School of Medicine, Baltimore, Maryland, USA

**Keywords:** *Streptococcus agalactiae*, Group B *Streptococcus*, Simplexa, ARIES, NAAT

## Abstract

We conducted a comparative evaluation of the FDA-cleared Simplexa GBS Direct and ARIES GBS molecular assays for the detection of *Streptococcus agalactiae* (Group B *Streptococcus*, GBS) in 386 prospectively collected, broth-enriched vaginal/rectal swab specimens. The sensitivity of each test was 96.2% and specificity was ≥98.7% when compared to a combined direct and enriched culture method using chromogenic culture medium. A total of four specimens were called positive by both molecular assays but negative by culture, likely representing specimens with a low burden of GBS in these specimens. Two specimens were reported positive by culture but negative by both molecular assays. One of these specimens demonstrated atypically colored colonies on chromogenic agar; the other yielded typically colored colonies only observed after broth enrichment. Our data demonstrate equivalent performance of Simplexa and ARIES molecular assays for the detection of GBS in clinical specimens.

**IMPORTANCE:**

Clinical laboratories often face decisions regarding which of the multiple available molecular platforms would best fit their needs based on cost, workflow, menu, and diagnostic performance. Therefore, objective clinical comparisons of similar molecular tests are valuable resources to aid these decisions. We provide a clinical comparison of two FDA-cleared tests to routine culture and to each other that can be used by clinical laboratories when determining which of the available molecular platforms would best fit their laboratory in terms of workflow, cost, and performance.

## INTRODUCTION

*Streptococcus agalactiae*, also known as Group B *Streptococcus* (GBS), is a Gram-positive β-hemolytic bacteria that asymptomatically colonize the human gastrointestinal and genitourinary tracts (1). GBS is also a formidable pathogen and a leading cause of severe and life-threatening neonatal infections such as sepsis, meningitis, and stillbirth (1, 2). The primary risk factor for GBS infection in the neonate is transmission from maternal colonization of urogenital tract during the birthing process. The prevalence of vaginal or rectal colonization in pregnant women ranges from 10% to 30% and can be intermittent, transitory, or persistent. It is estimated that up to 50% of pregnant women colonized with GBS will transmit the bacteria to the neonate, posing risk of these neonatal infections (3).

Given that the primary route of GBS acquisition and infection in neonates is direct contact with GBS in the mother's urogenital tract during delivery, pregnant women are universally screened for vaginal/rectal colonization with GBS at 36 through 37 weeks of gestation as recommended by the American College of Obstetricians and Gynecologists. In the event of a positive test result, mothers are generally administered intrapartum β-lactam antibiotics to reduce the risk of transmission and subsequent disease (1, 4). Prior to the adoption of screening and prevention guidelines, approximately 7,600 cases of GBS infection occurred in newborns annually. Adherence to the current guidelines has reduced the incidence of early-onset neonatal GBS infections, from 1.7 cases per 1,000 births in the 1990s to 0.2 cases per 1,000 births as of 2020 (5).

The current “gold standard” for detection of GBS relies on preliminary broth enrichment of the vaginal/rectal swabs using a selective media, such as Lim broth, followed by subculture onto blood agar plates, where GBS is identified based on colonial growth demonstrating the characteristic “soft” β-hemolysis zones, or onto chromogenic agar where β-hemolysis or other GBS-specific enzyme reacts with colorimetric substrate to yield pigmented colonies (4, 6). Importantly, between 5% and 8% of GBS isolates are non-hemolytic, leading to false-negative results when using standard culture-based methods of detection (7). Current screening recommendations also endorse the use of broth-enriched specimens as input for nucleic acid amplification test (NAAT)-based detection of GBS. Compared to culture-based methods, implementing NAAT for GBS detection leads to quicker results and the ability to identify non-hemolytic GBS isolates (7, 8). Additionally, polymerase chain reaction (PCR)-based methods are typically associated with increased sensitivity compared to culture-based methods, though assay sensitivity varies (7–9).

This study evaluated two FDA-cleared molecular assays, the DiaSorin Molecular Simplexa GBS Direct (DiaSorin, Stillwater, MN) and the Luminex ARIES GBS (Luminex - A Diasorin Company, Austin, TX), which are sample-to-answer real-time PCR assays for the *in vitro* qualitative detection of GBS nucleic acid from 18 to 24 h Lim broth enrichments of vaginal/rectal specimen swabs obtained from antepartum women. Results from each PCR were independently compared to the gold standard culture-based method as well as to each other.

## MATERIALS AND METHODS

### Collection of vaginal/rectal specimens

Dual Liquid Stuart vaginal/rectal swabs (BBL CultureSwab; BD, Sparks, MD) were collected during routine prenatal GBS screening from antepartum women and processed for routine standard of care culture (described below). Specimens used for research testing were de-identified prior to enrollment into the study using an institutional review board (IRB)-approved process. The collection and use of clinical specimens for this study were in accordance with site-specific IRB-approved protocols (IRB study number PRO00047245; Medical College of Wisconsin).

### Cultures for identification of GBS

Swab specimens were used to directly inoculate chromID Strepto B agar (BioMerieux, St. Louis, MO) and were then placed into 3 mL Lim broth (Remel, Lenexa, KS) for 16–24 h incubation at 35 ± 2°C. Following incubation, 10 µL of the broth-enriched specimens was streaked to a second chromID Strepto B agar plate and incubated 16–24 h at 35–37°C. The combination of direct plating and plating after broth enrichment is standard of care in our laboratory and has demonstrated superior sensitivity for the detection of GBS when compared to post-broth enrichment plating alone (10). Both “direct” and “enriched” chromID Strepto B agar plates were examined for the presence of characteristically colored pale pink to red colonies indicative of GBS. The presence of these colonies on either plate was sufficient to call the specimen positive for GBS. If there were atypical appearing colonies (e.g., faint coloration, small size, etc.), colonies were further confirmed using catalase (negative), Lancefield group B latex agglutination test, or MALDI-ToF MS (MALDI Biotyper compass 4.1; Bruker, Billerica, MA) as part of the standard of care protocol.

### Simplexa GBS Direct assay

The Simplexa GBS Direct assay is a diagnostic assay that uses primers and fluorescent probes to amplify GBS bacterial DNA and an Internal Control DNA (IC) in each specimen. The assay targets a conserved region of the *cfb* gene to identify *S. agalactiae* in the specimen and the IC is used to detect PCR failure and/or inhibition. Testing was performed following the package insert. Briefly, 50 µL of Lim broth-enriched specimen and 50 µL of Simplexa GBS Direct Reaction Mix were transferred to the sample and reaction wells, respectively, in the direct amplification disc. Specimen information was added to the instrument software and the disc was loaded into the LIAISON MDX (DiaSorin, Stillwater, MN). The total assay run time was 65 min and up to eight (8) specimens could be run simultaneously per amplification disc. Any specimens reported as invalid were retested once provided the enrichment broth was available. Positive and negative controls were run on weekly basis.

### ARIES GBS assay

The ARIES GBS assay targets a genomic region downstream from the *S. agalactiae cfb* gene and uses the Luminex Corporation's MultiCode real-time PCR chemistry in combination with ARIES systems (Luminex - A DiaSorin Company, Austin, TX). The ARIES system performs automated nucleic acid extraction and purification, PCR, and real-time detection of the target nucleic acid sequences. The package insert was followed for testing of the ARIES assay. Briefly, 200 µL of Lim broth-enriched specimen was transferred to the cassette sample chamber and the cassette was loaded on the ARIES instrument. The total assay run time was 1 h 58 min and up to six (6) specimens can be tested simultaneously. Any specimens reported as invalid were retested once provided the enrichment broth was available. Positive and negative controls were run on weekly basis.

### Statistical analysis

Results from the Simplexa GBS Direct assay and ARIES GBS assay were independently compared to the gold standard method, which was the standard of care culture result. Performance characteristics including sensitivity, specificity, overall agreement, and 95% confidence intervals were calculated using standard methods. Both the molecular devices were compared side by side to calculate positive percent agreement and negative percent agreement.

## RESULTS

A total of 386 prospective specimens were tested by Simplexa, ARIES, and culture. The culture-based prevalence of *S. agalactiae* was 13.7% (53/386). The initial rate of “invalid” results was 0.8% (3/386) for Simplexa and 4.9% (19/386) for ARIES. Two specimens initially reported as “invalid” by Simplexa were available for repeat testing; one was reported as negative upon retest while the other remained “invalid” resulting in a final success rate of 99.7% (384/385). Thirteen specimens initially reported as “invalid” by ARIES were available for repeat testing; three were reported as negative upon retest while the remaining ten were reported as “invalid” resulting in a final success rate of 97.4% (370/380). Of note, only one specimen was reported as “invalid” by both Simplexa and ARIES upon initial and repeat test.

When compared to culture, Simplexa and ARIES demonstrated equivalent sensitivity of 96.2% and specificity of ≥98.7% (Tables 1 and 2). Two specimens were reported positive by culture but negative by both Simplexa and ARIES. One of two specimens was negative upon direct plating to CHROMagar but demonstrated characteristic pink colonies when plated following broth enrichment, suggesting a very low concentration of *S. agalactiae* in the specimen. The other specimen demonstrated atypical appearing pink colonies on CHROMagar. Further testing of the isolate revealed a negative catalase reaction and a positive Lancefield group B latex agglutination test. These results are consistent with an identification of *S. agalactiae* but may not be specific to this species (see discussion). Four specimens were reported negative by culture but tested positive by both Simplexa and ARIES. No further characterization of these specimens was conducted.

**TABLE 1:** Performance of the Simplexa GBS Direct assay compared to culture

		Culture		
		Positive	Negative	Total
Simplexa GBS Direct	Positive	51	4^*a*^	55
	Negative	2^*b*^	327	329
	Total	53	331	384^*c*^
		Sensitivity: 96.2% (86–99)		
		Specificity: 98.8% (98–99)		

^*a*^ All four specimens were reported as positive by Simplexa and ARIES.

^*b*^ Both specimens were reported as negative by Simplexa and ARIES. One of two was culture positive only after Lim broth enrichment and demonstrated characteristic pink colonies on CHROMagar. The other had atypical appearing pink colonies on CHROMagar but was catalase negative and tested positive by Lancefield type B latex agglutination assay.

^*c*^ One specimen was initially reported as invalid and was unavailable for repeat testing. One specimen was reported as invalid upon initial and repeat test.

**TABLE 2:** Performance of the ARIES GBS assay compared to culture

		Culture		
		Positive	Negative	Total
Simplexa GBS Direct	Positive	51	4^*a*^	55
	Negative	2^*b*^	313	315
	Total	53	317	370^*c*^
		Sensitivity: 96.2% (86–99)		
		Specificity: 98.7% (97–99)		

^*a*^ All four specimens were reported as positive by Simplexa and ARIES.

^*b*^ Both specimens were reported as negative by Simplexa and ARIES. One of two was culture positive only after Lim broth enrichment and demonstrated characteristic pink colonies on CHROMagar. The other had atypical appearing pink colonies on CHROMagar but was catalase negative and tested positive by Lancefield type B latex agglutination assay.

^*c*^ Sixteen specimens initially reported as invalid either remained invalid after repeat test (*n* = 10) or were unavailable for repeat test (*n* = 6).

A head-to-head comparison of Simplexa and ARIES results demonstrated 100% overall agreement (i.e., equivalent positive and negative agreement) between the two tests (Table 3).

**TABLE 3:** Comparison of the Simplexa GBS Direct and ARIES GBS assays

		Simplexa GBS Direct		
		Positive	Negative	Total
ARIES GBS	Positive	54	0	54
	Negative	0	315	315
	Total	54	315	369^*a*^
		Positive Agreement: 100% (92–100)		
		Negative Agreement: 100% (98–100)		

^*a*^ Seventeen specimens failed to generate a valid result or were unavailable for repeat testing on Simplexa, ARIES, or both and were excluded from analysis.

## DISCUSSION

Antepartum screening for *S. agalactiae* colonization remains an essential component of current guidelines and has aided in reducing early-onset invasive neonatal infections (4). Incorporation of molecular tests to detect *S. agalactiae* in broth-enriched specimens can increase both sensitivity and specificity when compared to standard or chromogenic-based culture methods (7, 8, 11). Molecular assays including standard PCR, loop-mediated isothermal amplification, and helicase-dependent amplification-based tests are available for the detection of *S. agalactiae* and are available in on-demand and batch type instruments and workflows making them widely available to clinical laboratories. Factors including cost, level of complexity, existing instrumentation, and test volume are important considerations when selecting the test that best meets a laboratory's individual needs. Importantly, prior studies have demonstrated variable performance ranging from 90% to 98% overall agreement between NAATs using standard PCR and isothermal amplification technologies, including automated and manual workflows (7, 9, 11). Therefore, it is valuable to compare the performance of these tests when considering adoption or transition from an existing platform or modality.

In this study, we compared the performance of two PCR-based, sample-to-answer assays for the detection of *S. agalactiae* in Lim broth-enriched vaginal/rectal swab specimens obtained from pregnant females. Each molecular test was compared to a combination of direct and broth-enriched culture utilizing chromogenic medium. Simplexa and ARIES demonstrated equivalent performance when compared to culture (sensitivity of 96.2%; specificity of ≥98.7%).

Four specimens reported positive by both molecular tests that were negative by a combined direct and broth-enriched culture. These results likely represent specimens containing a low concentration of *S. agalactiae* rather than false-positive molecular results since each molecular assay targets a different genetic sequence. Though low concentration has been associated with reduced risk of early-onset infections (12), these specimens could also represent poorly collected or inappropriately transported specimens that resulted in reduced culture sensitivity. Assuming that these four specimens were falsely negative by culture, the use of molecular methods demonstrated 7.6% increase in positive results compared to direct and enriched cultures. Prior studies have demonstrated similar findings with PCR being equivalent or superior to culture, even when using chromogenic medium (7, 13).

Two specimens were reported positive by culture but negative by both molecular assays. The first was negative upon direct plating but demonstrated characteristic pink colonies on chromogenic agar when subcultured from Lim broth. This suggests a very low concentration of *S. agalactiae* in the broth, or a false-positive culture result. The second demonstrated *atypical* pink colonies on CHROMagar upon both direct and broth-enriched platings. Further characterization demonstrated a negative catalase reaction and a positive Lancefield group B latex agglutination result consistent with *S. agalactiae*. Importantly, chromogenic agars including ChromID StreptoB are reported to demonstrate specificity ranging from 73% to 98% (14, 15). This variable and comparatively reduced specificity can result from subjective visual assessment of colony color as well as cross-reactivity of other bacterial species including viridans group streptococci and *Enterococcus* that produce faintly colored or atypical appearing pink colonies on these medium. Because of this, it is recommended to confirm the identity of any atypical appearing colonies obtained on chromogenic medium (16). Our isolate was confirmed as catalase negative and positive for Lancefield group B antigen agglutination; however, *Streptococcus pseudoporcinus* and the recently described *Streptococcus halichoeri* have both been noted to be positive for Lancefield group B antigen (17, 18). Given these limitations, in conjunction with negative results from both Simplexa and ARIES, it is possible or likely that one or both specimens were truly negative for *S. agalactiae*. If not using a molecular test to identify *S. agalactiae* in broth-enriched specimens, we would recommend confirmation of atypically appearing colonies using alternative methods such as MALDI-ToF MS that are better able to discriminate species with positive Lancefield group B reactions.

The strengths of this study are the large “*n*” (386 specimens), prospective design, and use of a combined direct plus enriched culture method as gold standard. Study weaknesses include the single center design and incomplete resolution of discordant samples, though two independent molecular assays were in 100% agreement.

In conclusion, the Simplexa and ARIES molecular tests have proven equivalent performance and may be superior to the use of chromogenic medium for the detection of *S. agalactiae* in antenatal broth-enriched screening specimens. A notable advantage of molecular tests is increased sensitivity compared to culture-based methods, demonstrated by the detection of *S. agalactiae* in four (7.6%) additional culture-negative specimens, and increased specificity when compared to chromogenic medium.
